# The kidney and the balance of sulfur and nitrogen as fundamental components of pH homeostasis

**DOI:** 10.14814/phy2.70885

**Published:** 2026-05-18

**Authors:** Vincenzo Graziano, Antonio Angeloni, Paolo Mené, Andrea Bellelli

**Affiliations:** ^1^ Department of Molecular and Clinical Medicine Sapienza University of Rome and UOC Nefrologia e Dialisi, Sant'andrea Hospital Rome Italy; ^2^ Department of Wellbeing, Health and Environmental Sustainability Sapienza University of Rome and UOC Patologia Clinica, Policlinico Umberto I Rome Italy; ^3^ Department of Biochemical Sciences “A. Rossi Fanelli” Sapienza University of Rome Rome Italy; ^4^ Present address: Department of Medical and Surgical Sciences (DIMEC) Alma Mater Studiorum University of Bologna Bologna Italy

**Keywords:** acid–base balance, acidosis, alkalosis, ammoniagenesis, net acid excretion

## Abstract

The homeostasis of blood and tissue pH is fundamental for life, and pH imbalances may lead to coma and death. The regulation of the acid–base balance involves primarily the lungs and kidneys, and is strictly integrated via the endocrine and nervous systems. Crucial theoretical concepts have been developed to describe these functions, for example the Base Excess (BE) for the interpretation of the blood gas analysis or the Net Acid Excretion (NAE) for the interpretation of the kidney contribution to acid–base homeostasis. However, these concepts, coming from different research fields, have been insufficiently integrated. The present review collects information about the reciprocal correlation between respiratory and urinary parameters under physiological and common pathological conditions. A case in point will be made for metabolic acidoses, a heterogeneous group of disturbances whose interpretation would greatly benefit from the integration of information provided by the blood gas analysis with that provided by urinalysis, including the measurement of net acid excretion and urinary ammonium.

## Graphical abstract

Biochemical processes involved in ammoniagenesis and regulation of pH homeostasis.

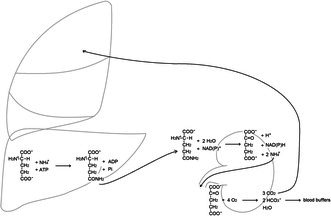

## 1 INTRODUCTION

In spite of the huge amount of information available on the subject of acid–base balance, a coherent description is still incomplete and often the excess of detail and the selective focus on the function of lung or kidney may obscure the global picture. Moreover, the acid–base balance may be challenged by a host of different pathophysiological conditions that may affect organs not directly involved in pH regulation, such as the gastrointestinal tract. Finally, the diet may impose a very variable acid or base load making the physiological ranges of pH‐related analytes in the urine very wide and of difficult diagnostic interpretation. The aim of this review is to select the most relevant information and to present it in a logically coherent framework. In this review, we privilege the analysis of information that can be gathered using minimally invasive measurements; many important details that help clarify the molecular mechanism of the functions here described but that are not measured in routine clinical analyses will be omitted.

## 2 DAILY PRODUCTION OF ACIDS AND BASES

Our diet provides, and our metabolism produces, significant amounts of acids and relatively little bases, both of which must be disposed of in order to maintain the pH homeostasis. The most abundant body acid is CO_2_ that our metabolism produces at a rate of over 14 mol/day or 10 mmol/min. CO_2_ as such is disposed of entirely by the lungs.

Depending on the protein abundance in our diet, we produce a significant amount of ammonia; indeed, with a recommended daily intake of 0.8 g protein/Kg body weight, an adult may be estimated to absorb at least 0.7 moles of nitrogen daily. Ammonia is a weak base that at physiological pH is almost completely protonated to ammonium ion; however, it is rapidly converted by the liver into nonbasic compounds like urea and glutamine, and only a minimal fraction enters the circulation.

Proteins contain sulfur in the amino acids Ser and Met; this element is converted to sulfuric acid and excreted by the kidney, at a rate of approx. 30 mEq/day. Due to the presence of sulfur and other acidic components, the net total noncarbonic acid introduced with our diet has been estimated at approx. 50 mEq/day (Kurtz, 2018; Remer & Manz, 1995). The diet also contains phosphate salts and bicarbonate that behave as bases, but usually they are exceeded by noncarbonic acids.

The hydrogen ion is never present as such in solution, as it is always bound to other compounds that behave as bases; the most important such compounds in the blood and intra‐ and extra‐cellular fluids are His residues on plasma proteins, the conjugate bases of weak acids (e.g. bicarbonate and phosphate), and ammonia (as ammonium ion, NH_4_^+^). This causes the concentration of hydronium ions, H_3_O^+^, to be very low, that is, submicromolar, except in the gastric juice. Thus, it is safe to say that hydrogen ions produced by our metabolism are always bound to, and exchanged among, different buffers, and excretion of hydrogen ions is always accompanied by the excretion of other substances. The pH of body fluids is determined by the chemical reactions of its buffers, whereas the content of titratable acid is mainly determined by the buffers' concentration. Throughout this review we shall indicate the hydrogen ion as bound to buffer compounds, and shall represent these compounds in their most common protonation state at physiological pH. All buffers present in any given compartment exchange hydrogen ions to reach a common equilibrium condition (so‐called isohydric principle); thus any weak base can be exchanged with any other, and the same may be said of any weak acid (see below, Equation 1 for an example). Because of the exchange of hydrogen ions among different buffers, metabolic production of a base is equivalent to consumption of an acid and vice versa.

### 2.1 Production, transport, and elimination of CO_2_

CO_2_ is produced by the oxidative catabolism of nutrients at a rate that depends on the energy requirements of the organism, and amounts to over 14 mol/day. It cannot be stored in the organism and the rate of respiratory elimination matches that of production. CO_2_ behaves as a weak acid, but is produced by oxidation of nutrients that are not themselves acidic, for example, sugars; thus the daily acid load does not depend on the actual acidity of the food.

The amount of CO_2_ produced in a couple of hours (in the order of 1.2 mol) would exceed the buffer capacity of the whole human body (estimated at 0.75–1.0 mol from the data of Siggaard‐Andersen & Fogh‐Andersen, 1995) without its rapid respiratory elimination. The total content of CO_2_ in blood, approx. 20–22 mM (and 25–27 mM in plasma), largely exceeds the gas solubility and is made possible by the blood buffers that promote its acidic dissociation:

(1) $${\mathrm{CO}}_{2}+H_{2}O+X⇆{{\mathrm{HCO}}_{3}}^{-}+{\mathrm{XH}}^{+}$$

where *X* represents the mixture of noncarbonic blood buffers, mainly constituted by His residues of blood proteins (Bellelli, 2025).

**FIGURE 1:**
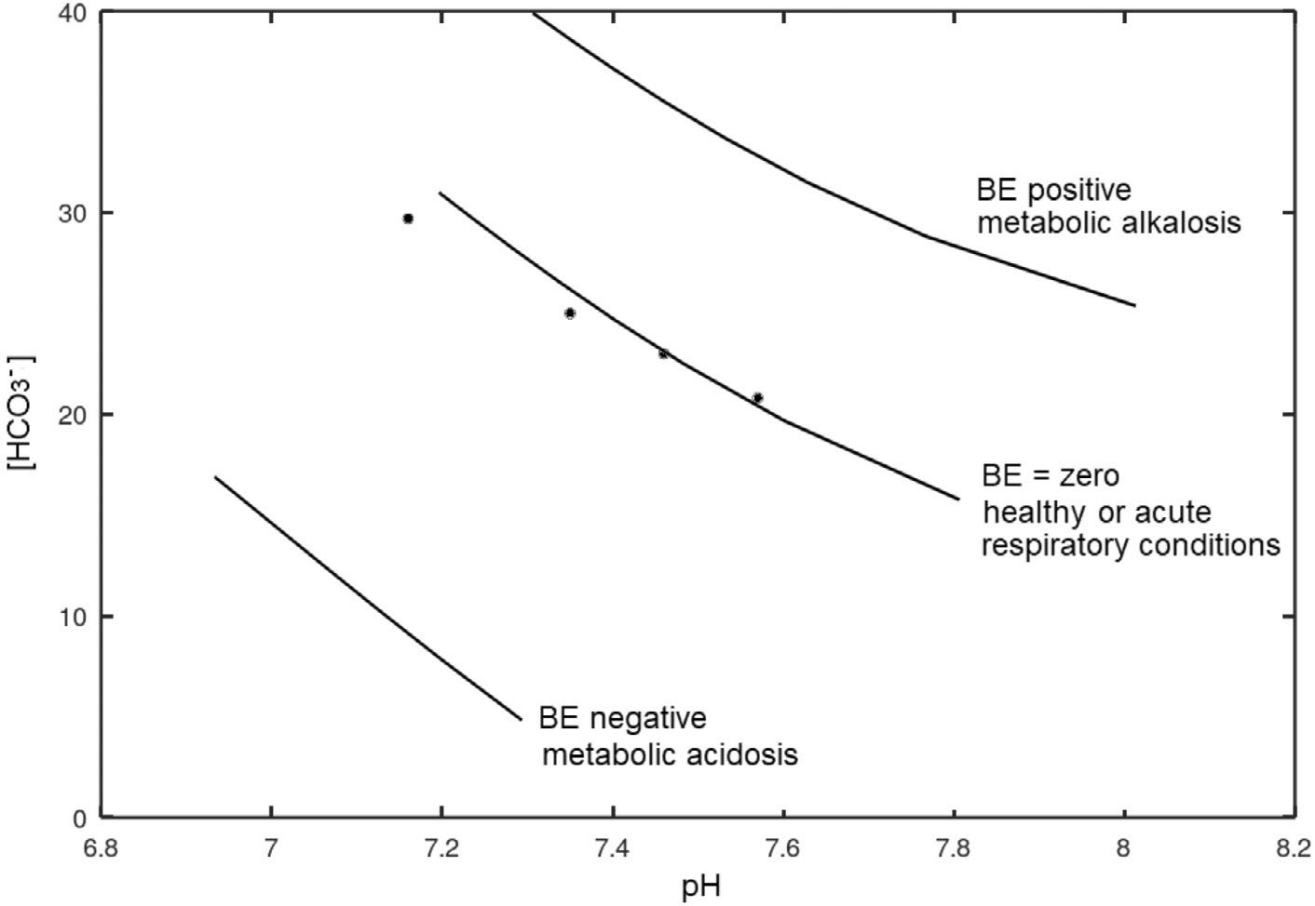
Davenport diagram showing the relevance of the *S*_dp_ parameter. Each line in the graph describes the titration of a blood sample with PCO_2_; the points are experimental data taken from Davenport (1974). The model used to generate the lines is described elsewhere (Bellelli, 2025); briefly, Equation (1) makes it obvious that during the transport of CO_2_ from the tissues to the lungs, as well as during in vitro titrations with CO_2_, the sum of bicarbonate and the other deprotonated components of blood buffers is constant; we call this value *S*_dp_: *S*_dp_ = [HCO_3_^−^] + [X]. The lung can only excrete CO_2_, that is, bicarbonate combined with hydrogen ions: In no way can it separate these two ions; thus, the lung cannot change the *S*_dp_. The organ that selects which ions must be coupled to hydrogen ions for excretion, and thus can change the *S*_dp_, is the kidney. The stomach is another organ capable of separating hydrogen ions (secreted in the gastric juice with chloride) from bicarbonate ions (reabsorbed in the serum) and vomiting causes metabolic alkalosis. In order to obtain the lines shown in the figure, arterial blood from the patient is submitted to a standard gas analysis to measure the parameters pH and PCO_2_; the total concentration of noncarbonic buffers (Xt = [*X*] + [XH^+^]) is estimated from the hemoglobin content using standard formulas (Siggaard‐Andersen & Fogh‐Andersen, 1995). From these parameters one derives *S*_dp_ and the effect of PCO_2_ on pH and bicarbonate concentration.

Given the high buffer capacity of blood, 90% of total CO_2_ is accounted for by the nonvolatile bicarbonate anion, 5% by protein carbamates and only 5% by CO_2_ as such. Reaction 1 proceeds from left to right in the tissue capillaries, where CO_2_ is released, and from right to left in the pulmonary capillaries, where CO_2_ is excreted. Equation (1) shows that the sum of deprotonated buffer components (*S*_dp_ = [HCO_3_^−^] + [*X*]) is constant and may be used to predict the effects of changes of PCO_2_ (see Figure 1) (Bellelli, 2025).

### 2.2 Ammoniagenesis by the kidneys and its role in acid–base balance

Nitrogen is introduced with the diet, mainly as protein amino acids, and as NH_4_^+^ produced by the intestinal microbiota. The metabolism of nitrogen affects the body buffers in several ways; for example, in the case of alanine one ammonium ion is generated together with bicarbonate:

(2) $$C_{3}H_{7}O_{2}N+3\;O_{2}\to 2\;{\mathrm{CO}}_{2}+{{\mathrm{HCO}}_{3}}^{-}+{{\mathrm{NH}}_{4}}^{+}+H_{2}O$$

These two ions are immediately converted to urea by the liver:

(3) $$2\;{{\mathrm{NH}}_{4}}^{+}+2\;{{\mathrm{HCO}}_{3}}^{-}\to \mathrm{CO}{\left({\mathrm{NH}}_{2}\right)}_{2}+{\mathrm{CO}}_{2}+3\;H_{2}O$$

Equation (2) and (3) show that acids and bases are produced and consumed together; thus, disposal of NH_4_^+^ via liver urea cycle consumes all the HCO_3_^−^ (or base equivalents) that were obtained during its production (Equation 2).

Another possible usage of ammonia by the liver is the biosynthesis of glutamine catalyzed by glutamine synthase:

(4) $$C_{5}H_{8}O_{4}N^{-}+{{\mathrm{NH}}_{4}}^{+}\to C_{5}H_{10}O_{3}N_{2}+H_{2}O$$

Nitrogenous compounds are eliminated by the kidney in the form of urea, ammonium ion, creatinine, and less relevant others. The renal elimination of ammonia exceeds the amount of this compound carried by the blood (<0.04 mM) and requires kidney ammoniagenesis, which relies on two consecutive deaminative reactions of glutamine, catalyzed by glutaminase and glutamate dehydrogenase, respectively:

(5) $$C_{5}H_{10}N_{2}O_{3}+H_{2}O\to C_{5}H_{8}{{\mathrm{NO}}_{4}}^{-}+{{\mathrm{NH}}_{4}}^{+}$$

(6) $$\begin{array}{c} C_{5}H_{8}{{\mathrm{NO}}_{4}}^{-}+H_{2}O+\mathrm{NAD}{\left(P\right)}^{+}\to C_{5}H_{4}{O_{5}}^{-2}\\ \;+{{\mathrm{NH}}_{4}}^{+}+\left[\mathrm{NAD}\left(P\right)H+H^{+}\right] \end{array}$$

Notice that when a hydrogen/hydronium ion is produced in a redox reaction that stores electrons in a cofactor, it is disposed of in other redox reactions or by the respiratory chain in the mitochondrion and does not participate in the pH homeostasis (hence it is indicated in square brackets, together with its reduced cofactor). Glutamine consumption by the kidney is very significant and is sustained by liver biosynthesis (Equation 4); under some pathological conditions, it may lead to the degradation of muscle protein and muscular wastage. Consistently, Gln is the most abundant free amino acid in the serum at a concentration of 420–700 𝜇Mol/L (Blaauw et al., 2020).

The 2‐ketoglutarate produced by the above reactions is further metabolized by the kidney itself (or released in the blood as lactate and metabolized by the liver). The overall reaction (excluding redox cofactors) is as follows:

(7) $$C_{5}H_{4}{O_{5}}^{-2}+4\;O_{2}\to 3\;{\mathrm{CO}}_{2}+2\;{{\mathrm{HCO}}_{3}}^{-}+H_{2}O$$

As long as the acid–base equilibrium is concerned, the overall balance of reactions (5), (6), (7) is production of 2 equivalents of NH_4_^+^, of which, on average, one is excreted in the urine, while the other is reabsorbed in the blood, and carried to the liver for conversion to urea (Equation 3) and 2 moles of bicarbonate that are reabsorbed in the blood (Bourgeois & Houillier, 2024; Kurtz, 2014). Thus, for every ammonium ion excreted in the urine, one bicarbonate ion is donated to the blood and contributes to increase the *S*_dp_, and to raise the blood pH.

Renal ammoniagenesis amounts to 60–80 mMol/day but can increase to 300–400 mMol/day in nonrenal metabolic acidosis (Weiner et al., 2015). Since approx 50% of ammonia is reabsorbed in the blood, the urine content of ammonia in a healthy adult averages 30–40 mmol/day, but can increase up to 150–200 mMol/day (Uribarri et al., 2022). Unfortunately, urinary ammonia is not routinely measured by most laboratories, and may be affected by false positive results caused by the possible contamination by urease‐producing bacteria; an imperfect substitute is the Urine Anion Gap (uAG) (Batlle et al., 2017, 2025), defined as:

$$\mathrm{uAG}=\left(\left[{\mathrm{Na}}^{+}\right]+\left[K^{+}\right]\right)-\left(\left[{\mathrm{Cl}}^{-}\right]+\left[{{\mathrm{HCO}}_{3}}^{-}\right]\right)$$

The uAG is a parameter of uncertain interpretation because its value is determined by several unmeasured positive and negative ions (Kirschbaum et al., 1999; Uribarri & Oh, 2021); however, a strongly negative uAG may be suggestive of increased ammonium excretion (Batlle et al., 2025), an indication to be confirmed by direct measurement of urinary ammonium and NAE.

### 2.3 Regulation of acid excretion by the kidneys

The net acid excretion (NAE) is given by the sum of H^+^ bound to ammonium and other buffers, minus the bases (bicarbonate) (Remer & Manz, 1995):

$$\begin{array}{c} \mathrm{Net}\;\text{Acid Excretion}\;\left(\text{urine}\right)\\ \;={{\mathrm{NH}}_{4}}^{+}+\left(H_{2}{{\mathrm{PO}}_{4}}^{-}\;\text{and other titratable acids}\right)-{{\mathrm{HCO}}_{3}}^{-} \end{array}$$

**FIGURE 2:**
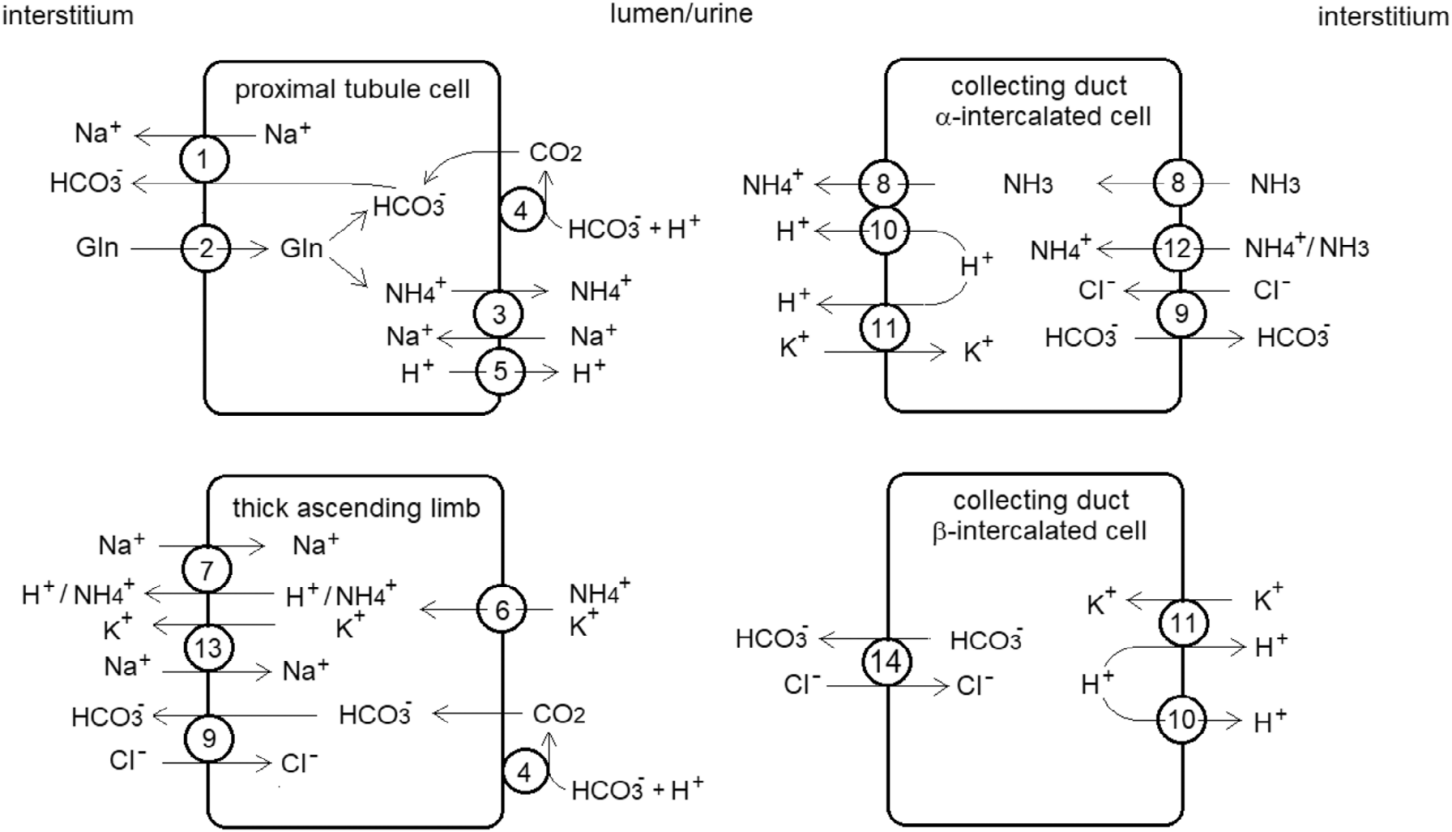
Schematic representation of the major renal transporters involved in acid–base regulation (redrawn from Weiner & Verlander, 2017; Imenez Silva & Mohebbi, 2022; Palmer et al., 2021; Bourgeois & Houillier, 2024). Transporters: 1: NBCe1; 2: SN1; 3: NHE3; 4: Carbonic anhydrase IV; 5: H^+^ ATPase; 6: NKCC2; 7: NHE4; 8: RhCG; 9: AE1 (band 3); 10: H^+^ ATPase; 11: H^+^/K^+^ ATPase; 12: RhBG; 13: Na^+^/K^+^ ATPase; 14: Pendrin. Direct secretion of H^+^ in the urine occurs mainly in the proximal tubule and in the collecting ducts and is operated by specific isoforms of multimeric vacuolar H^+^‐ATPases (Karet et al., 1999; Finberg et al., 2005; Ayasse et al., 2024), and H^+^/K^+^ ATPases (Gumz et al., 2009). H^+^ are donated by intracellular buffers, which in turn exchange them with extracellular buffers; thus urinary excretion of H^+^ raises the *S*_dp_.

In healthy subjects the pH of urine ranges between 4.5 and 7, and is mainly governed by the phosphate and urate buffers, whose protonation state changes over the physiological pH range of urine, their pKa being within the physiological pH range of urine. The ammonium ion is the most important component of NAE, but is always in the same protonation state because its pKa lies well outside the pH range of urine; thus it behaves as a “fixed” or “strong” ion, and has little direct effect on the urine pH. H^+^ and NH_4_^+^ are selectively transported from the blood/interstitium to the urine and vice‐versa; the major renal transporters are listed in Table 1 and their location is schematically depicted in Figure 2.

**TABLE 1:** Principal renal transporters involved in the acid–base regulation.

Transport	Transporter	Regulated by
Urine acidifiers		
H^+^ from interstitium/ blood to urine	H^+^ ATPase (collecting ducts) (Brown et al., 1988)	Positively regulated by acidosis and hypokalemia. RhCG increases activity of H^+^ ATPase (Bourgeois et al., 2018)
H^+^ from interstitium/ blood to urine (and K^+^ in the opposite direction)	H^+^ / K^+^ ATPase (collecting duct) (Gennari, 2011)	Positively regulated by acidosis and aldosterone
NH_4_^+^ secretion in the urine	NHE3 transporter (proximal tubulus); RhBG (collecting ducts) (Harris et al., 2023; Weiner & Verlander, 2017; Bourgeois et al., 2018)	Acidosis promotes the biosynthesis of the enzymes required for NH_4_^+^ production (mainly glutaminase)
NH_3_	RhCG (collecting ducts; works in parallel with H^+^ ATPase) (Biver et al., 2008)	As for NH_4_^+^
Creation of medullary NH_4_^+^ gradient	NKCC2 (Henle's loop) (Weiner & Verlander, 2017)	Expression of NKCC2 increases in metabolic acidosis (Weiner & Verlander, 2017)
HCO_3_^−^ from urine to interstitium/ blood	Na^+^/HCO_3_^−^ cotransporter (NBCe1; proximal tubulus) AE1 Cl^−^/HCO_3_^−^ exchanger (SLC4A1; collecting duct) (Palmer et al., 2021)	
Urine alkalinizers		
HCO_3_^−^ from interstitium/ blood to urine (and Cl^−^ in the opposite direction)	Na^+^‐independent (pendrin, SLC26A4) and Na^+^‐driven (SLC4A9/A8) Cl^−^/HCO_3_^−^ exchangers in—intercalated cells of the distal nephron (Vitzthum et al., 2024)	HCO_3_^−^ excretion negligible if urine pH is <6.6; it is positively regulated by secretin (Berg et al., 2024)
CO_2_ (formed because of acidification of bicarbonate) from urine to interstitium/ blood	Aquaporin 1 (proximal tubule) (Wang et al., 2024)	
Amphoteric (true buffers; can accept or donate hydrogen ions)		
Phosphate (600–1500 mg P/day)	Partially reabsorbed by the proximal tubule by sodium phosphate cotransporters (NaPi‐2a, NaPi‐2c, PiT‐2) (Lederer, 2014)	Tubular reabsorption of phosphate is regulated by PTH and vitamin D, along with fibroblast growth factor‐23 (FGF‐23).

**FIGURE 3:**
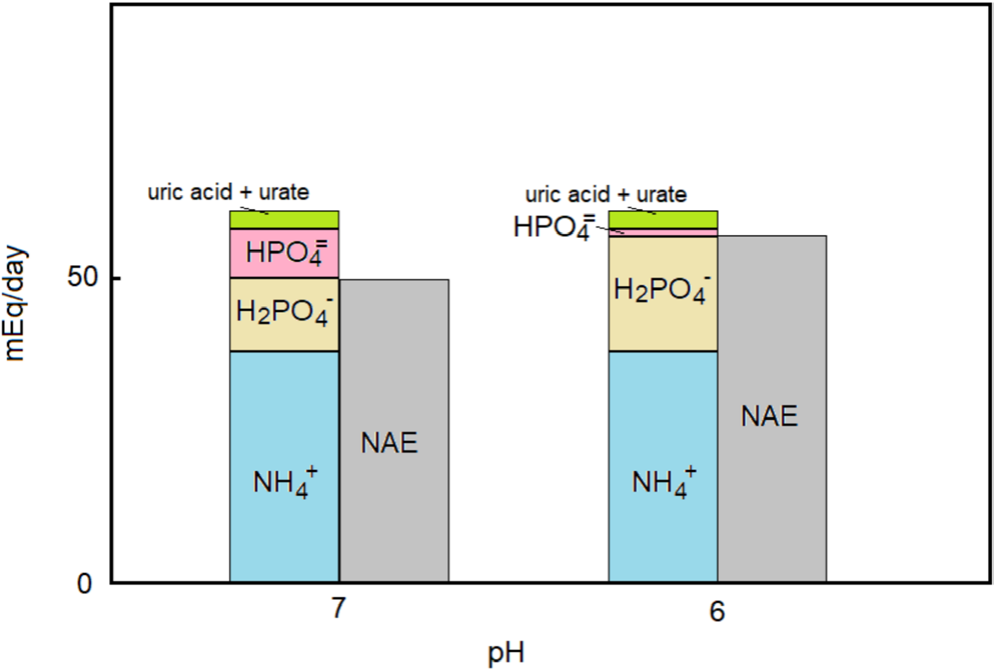
Effect of pH on urine buffers and NAE. This example considers a urine sample presenting: Volume 1500 mL/day; pH = 7; [NH_4_^+^+NH_3_] = 38 mmol/day; [H_2_PO_4_^−^ + HPO_4_^−2^] = 20 mmol/day; [uric acid+urate] = 3 mmol/day. In this sample the NH_4_^+^ accounts for 99.4% of total ammonia, H_2_PO_4_^−^ for 61% of total phosphate, and uric acid for 2% of total urate (values calculated using the following estimates of pKa: 9.25; 7.2; and 5.3 respectively). The NAE of this sample is 49.4 mEq/day. Let us now consider a urine sample of identical composition, except that the collecting tubules have secreted more H^+^, lowering pH to 6; the percentages given above will all increase to 99.9%, 94%, and 17% respectively; NAE increases by 7.1 mEq/day to 56.3 mEq/day. In this example, NH_4_^+^ contributes 76% of NAE at pH = 7 and 67% of NAE at pH = 6; over 90% of the increase of NAE is accounted for by the increased degree of protonation of phosphate; the increase in free H^+^ and in the degree of protonation of ammonia is negligible; that of urate is significant but, due to the low absolute concentration of this compound, its quantitative buffering capacity is modest.

The relationship between NAE and urine pH rests mainly on the titration of true buffers (i.e. compounds whose pKa lies within the pH range of urine). This is best explained with an example, as shown in Figure 3.

The above example demonstrates that ammonium may be the major determinant of NAE, but is not the major determinant of urine pH (Weiner & Verlander, 2017). This is consistent with older observations showing that the relationship between the logarithm of ammonium concentration and pH in the urine is linear with a slope of −0.15 to −0.25 (Cholnoki, 1970), much smaller than the value expected for a solution of ammonium chloride. A comparison of our theoretical example with the actual data by Bobulescu et al. (2013) and by Uribarri et al. (1995) is reported in Table 2.

**TABLE 2:** Elimination of ammonia and other acids by the kidney under healthy and selected pathological conditions.

	Control group^a^	Urine acid stone formers^a^	Type 2 diabetes mellitus^a^	Control group^b^
Urine pH	5.92 ± 0.40	5.49 ± 0.31	5.41 ± 0.25	—
NH_4_^+^ (mEq/day)	36 ± 8	33 ± 13	38 ± 6	35 ± 3
NAE	44 ± 15	57 ± 16	62 ± 16	48 ± 6
NH_4_^+^/NAE	90%	60%	60%	
Phosphate (mmol/day)	19	23	19	22
Urate (mmol/day)	3.2	2.9	3.3	—
Na^+^ (mEq/day)				126.5 ± 37
K^+^ (mEq/day)				54 ± 4
Cl^−^ (mEq/day)				130 ± 12
Sulfate (mEq/day)				34 ± 2
Urinary AG				+ 51

^a^ Bobulescu et al. (2013).

^b^ Uribarri et al. (1995).

It has been demonstrated that acidosis, and particularly metabolic acidosis, promotes an increase in the biosynthesis of kidney glutaminases, and hence of ammoniagenesis (Tong et al., 1986); this is the most relevant linkage between blood pH and NAE.

Bicarbonate is not an important buffer in the urine, because filtered HCO_3_^−^ is almost entirely reabsorbed, except in pathological conditions, and its concentration in the urine is negligible if pH <6.6 (Batlle et al., 2025). However, it has recently been demonstrated that renal excretion of HCO_3_^−^ may be stimulated by the hormone secretin, previously associated mainly to the regulation of pancreatic secretion (Berg et al., 2021), and mice in which the secretin receptor was knocked out developed chronic metabolic alkalosis (Berg et al., 2024). In fact, under those conditions in which renal excretion of HCO_3_^−^ occurs, it alkalinizes the urine and acidifies the blood by reducing the *S*_dp_.

### 2.4 Sulfuric acid and other diet‐derived acidic and basic substances

The main catabolic pathway of sulfur‐containing amino acids (Cys and Met) produces sulfuric acid, which immediately dissociates into sulfate and H_3_O^+^ (buffered by bicarbonate and other blood buffers). The overall reaction of Cys catabolism is as follows:

(8) $$\begin{array}{c} C_{3}H_{7}{\mathrm{NSO}}_{2}+4\;½\;O_{2}+{{\mathrm{HCO}}_{3}}^{-}\to 4\;{\mathrm{CO}}_{2}+{{\mathrm{NH}}_{4}}^{+}\\ \;+{{\mathrm{SO}}_{4}}^{-2}+2\;H_{2}O \end{array}$$

where the nonacidic thiol group of Cys (pKa ≃ 9) is converted to the entirely dissociated sulfuric acid. The hydrogen ions released by sulfuric acid are buffered by HCO_3_^−^ or the other blood buffers. Comparing the catabolism of Ala (Equation 2) with that of Cys (Equation 8), we observe that the former yields one HCO_3_^−^, whereas the latter consumes one HCO_3_^−^.

Cys and Met represent approximately 3–6% of dietary amino acids; thus, a normal diet containing 0.8 g/kg. day of proteins provides approximately 15–40 mmol/day of sulfur (Passafiume et al., 2023), which are mainly metabolized to sulfate at a rate of 30–80 mEq/day and impose an equivalent acid load on blood buffers and ultimately on the kidney. Thus, under healthy conditions, sulfate and ammonium excretion may be approximately equivalent (Table 2).

Sugars and fats, as well as the carbon backbone of amino acids, are metabolized to CO_2_, thus they do not contribute to the nonvolatile acid load. However, some foods may contain organic acids or their conjugate bases, as well as alkaline or acidic phosphate salts, thus they may contribute to increase or decrease the net dietary acid load.

## 3 PATHOPHYSIOLOGY OF COMMON ACID–BASE DISTURBANCES OF RESPIRATORY ORIGIN

### 3.1 The assessment of the different components of pH imbalances

The respiratory component of pH imbalances is unequivocally estimated via the PCO_2_, which measures the gas gradient required by the lung to excrete all the CO_2_ produced by our metabolism, and increases in all disturbances causing reduced ventilation or impaired gas diffusion. The metabolic component of acid–base imbalances is best estimated by the Base Excess (BE) or Standard Base Excess (SBE) (Siggaard‐Andersen & Fogh‐Andersen, 1995), but this parameter gives no clue about the cause of the underlying disorder. A related problem is to estimate the extent of pathological and compensatory effects for the two components of acid–base imbalances; this is best standardized for PCO_2_ and bicarbonate, for which extensive empirical evidence has been gathered.

### 3.2 Respiratory acidosis

Acute onset impairment of ventilation or gas diffusion through the alveolar membrane causes an increase of PCO_2_ and a significant decrease of blood pH, coupled to a minor increase of bicarbonate; this triad of symptoms is the hallmark of acute respiratory acidosis. In pure acute respiratory acidosis the *S*_dp_ is unchanged and BE is zero (Table 3). As a response to acute respiratory acidosis, the kidneys increase elimination of acids in the form of ammonium (excreted in the urine together with chloride) and production of bicarbonate via increased ammoniagenesis (Adrogué & Madias, 1986; Abdulnour‐Nakhoul et al., 2019). Within a few days, the kidney response converts the acute respiratory acidosis into its chronic counterpart, characterized by the triad strongly increased PCO_2_, strongly increased bicarbonate, and moderately decreased blood pH. Increase of bicarbonate is paralleled by increased *S*_dp_ and positive BE. In chronic respiratory acidosis renal ammoniagenesis returns to normal levels, because the observed hypercapnia reflects the increased PCO_2_ gradient that the lungs require in order to eliminate the metabolically produced CO_2_, rather than an increased rate of production or a decreased rate of elimination of CO_2_, and once the kidneys have raised bicarbonate to its optimum compensatory concentration the system reaches a new steady‐state condition. Moreover, the near normalization of blood pH removes the stimulus that causes increased ammoniagenesis (Tannen, 1983). This is at variance with the majority of chronic metabolic acidoses, in which a new steady‐state cannot be reached because new acid is continuously produced, and a continuous and sustained increase of ammoniagenesis and acid excretion by the kidneys is required.

**TABLE 3:** Differential diagnosis of acid–base disturbances; relationships between NAE, urinary ammonia and base excess.

Type	Pathophysiology	Clinical findings: Urine	Clinical findings: Serum
Acute respiratory acidosis	Reduced ventilation and/or compromised gas exchange in the alveoli	Increased ammonia and NAE	Increased PCO_2_; moderately increased bicarbonate; severely decreased pH; BE = 0
Chronic respiratory acidosis	Same as in the acute conditions	When full compensation is reached ammonia and NAE return to normal values	Increased PCO_2_; strongly increased bicarbonate; moderately decreased pH; positive BE
Acute respiratory alkalosis	Hyperventilation (e.g. because of anemia or neurological lesions)	Decreased ammonia and NAE; bicarbonate may appear in the urine	Decreased PCO_2_; moderately decreased bicarbonate; severely increased pH; BE = 0
Chronic respiratory alkalosis	Same as in the acute condition	When full compensation is reached ammonia and NAE return to normal values	Decreased PCO_2_; strongly decreased bicarbonate; moderately increased pH; negative BE
Renal tubular acidosis TYPE 1—distal	Inability of the distal and collecting tubuli to secrete H^+^	pH usually >5.5; reduced NAE and urine ammonia	[HCO_3_^−^] usually 14–20 mM; normal sAG; BE negative
Renal tubular acidosis Type 2—proximal	Inability of the proximal tubuli to reabsorb HCO_3_^−^.	pH usually <5.5; presence of bicarbonate in the urine; reduced NAE and urine ammonia	[HCO_3_^−^] may be <10 mM; frequently associated to K^+^ wasting and hypokalemia; normal sAG; BE negative
Renal tubular acidosis Type 3—mixed	Both proximal and distal (rare)	A mix of the findings of types 1 and 2	A mix of the findings of types 1 and 2
Renal tubular acidosis Type 4—hyperkalemic	Aldosterone deficiency or resistance	Reduced NAE and urine ammonia; decreased ratio serum to urinary K^+^ (Kuo et al., 2011)	Reduced bicarbonate; hyperkalemia; normal sAG; BE negative
Acidosis in the course of chronic kidney failure	Reduced GFR; reduced elimination of nonvolatile acids; reduced ammoniagenesis	Decreased urinary ammonia; positive uAG; decreased GFR	Reduced bicarbonate; high sAG (HAGMA); BE negative
Diabetic ketoacidosis	Excess production of ketoacids	Increased urinary ammonia; increased NAE; pH <5.5; GFR not primarily affected	Reduced bicarbonate; high sAG (HAGMA); BE negative
Lactic acidosis	Excess production of lactic acid (sepsis, tissue hypoxia, etc.)	Increased urinary ammonia; increased NAE; pH <5.5; GFR not primarily affected	Reduced bicarbonate; high sAG (HAGMA); BE negative
Severe diarrhea	Loss of bicarbonate	Increased urinary ammonia; increased NAE; GFR not primarily affected	Reduced bicarbonate; high sAG (HAGMA); BE negative
Extra‐renal metabolic alkalosis	Increased production of bicarbonate (e.g. because of vomiting)	Alkaline urine; excretion of bicarbonate in the urine; reduced NAE	Increased bicarbonate; positive BE
Salt loosing tubulopathies	Several variants known	Alkalinization of urine possible (Kamel et al., 1998)	Increased bicarbonate; positive BE; usually hyperchloremic and hypokalemic

The correlation between the progressive increase of BE and the transient increase of ammonium excretion was confirmed by an interesting study on patients developing respiratory acidosis after cardiac surgery (Roehrborn et al., 2017); unfortunately, the number of patients was small and further data are required to fully characterize the extent and time evolution of the correlation.

### 3.3 Respiratory alkalosis

Respiratory alkalosis occurs whenever the patient increases his or her breathing rate (hyperpnea). Causes of hyperpnea are usually linked to hypoxia or reduced O_2_ content of blood: for example, adaptation to high altitude, anemia, hemoglobinopathies, neurological lesions, etc. If the condition is acute, the consumption of bicarbonate is modest and the pH increase is significant; *S*_dp_ does not change and BE is zero. If the condition is long‐standing, the kidney reduces ammoniagenesis and production of bicarbonate, which may appear in the urine; this normalizes the [HCO_3_^−^] / PCO_2_ ratio and moderates the pH increase. Chronic respiratory loss of CO_2_ promotes conversion of bicarbonate to CO_2_ and, coupled to renal losses, causes the *S*_dp_ to decrease and BE to become negative (base deficit). The renal response to acute respiratory alkalosis is transient (as in respiratory acidosis) and surprisingly rapid: in a study of simulated acclimation to high altitude up to 2800 m, the increase of urine pH from 6.0 up to 6.7 was reached in 6 h and declined to 6.2 in 24 h. The increase of urine pH was associated with an increase in urinary bicarbonate from 1.5 mM up to 9 mM in 6 h, again with a significant decline at 24 h (Ge et al., 2006); unfortunately the NAE was not measured in that study.

## 4 ACID–BASE IMBALANCES OF METABOLIC ORIGIN

The so called “metabolic” disturbances of the acid–base homeostasis, make up a heterogeneous group of disorders, whose cause may reside in dysfunction of the kidney, or in true disorders of metabolism, or in losses of acids or bases via other routes (e.g. vomiting, diarrhea). Moreover, within each group, one must take into account that unmeasured components may participate to the acid–base equilibrium; these are revealed by alterations in the serum anion gap (sAG), and should prompt further investigation in order to identify the unmeasured component. Measurement of urinary ammonia and serum sulfate may provide important diagnostic clues.

### 4.1 Metabolic acidoses

Metabolic acidosis (MA) presents low arterial blood pH; reduced serum HCO_3_^−^; decreased PCO_2_ (because of compensatory hyperventilation); diminished *S*_dp_; and negative BE (base deficit). The sAG may be normal or increased. MAs are strongly heterogeneous and obey different pathophysiological mechanisms; thus, they require further classification according to whether the observed MA is of renal or extra‐renal origin.

Extra‐renal MAs may recognize essentially two different pathogenetic mechanisms: (i) excess loss of bicarbonate; or (ii) metabolic production of excess nonvolatile acids. In both cases NAE and ammonia excretion are strongly increased because of a compensatory response of the kidney. By contrast in renal MAs NAE and ammonia excretion are decreased or insufficiently increased because the kidney, instead of compensating for a problem originating elsewhere, is itself the cause of the disturbance. It is thus apparent that urinary ammonium and NAE are important diagnostic parameters.

In Normal Anion Gap MAs (NAGMAs), the decrease in [HCO_3_^−^] is matched by an equal increase in [Cl^−^], hence the acidosis is hyperchloremic, whereas in High Anion Gap MAs (HAGMAs) unmeasured anions are increased.

### 4.2 Acidosis due to chronic kidney failure

The most important reasons of MA in chronic kidney disease (CKD) are: (i) the glomerular filtration rate decreases and becomes insufficient to remove the nonvolatile acids introduced with the diet, causing their concentration in the blood to increase; and (ii) the capacity for renal ammoniagenesis and distal tubular hydrogen ion secretion is impaired, leading to a reduction or at least an insufficiently increased NAE (Vallet et al., 2015). This acid retention may affect the buffering capacity of bone and muscle, which serve as major reservoirs for hydrogen ions, contributing to renal osteodystrophy, and to the possible disruption of insulin signaling (Korus et al., 2025). The acidosis in CKD entails an increased sAG due to retention of sulfate and other anions normally removed by the kidney. Reduced *S*_dp_ and negative BE complete the picture.

An interesting study by Uribarri et al. (1995) quantifies the relationship between daily urinary excretion of ammonia and sulfate, NAE, and the glomerular filtration rate (GFR). The values for severe CKD patients from this study are as follows: serum bicarbonate = 20.4 mM; GFR = 19 mL/min.; urinary ammonia = 19 mEq/day (value for healthy subjects: 35 mEq/day); urinary sulfate 26 mEq/day (value for healthy subjects: 34 mEq/day); NAE = 30.8 mEq/day (value for healthy subjects: 48 mEq/day). Unfortunately, arterial blood gas analyses were not collected in this study.

### 4.3 Renal tubular Acidoses

Renal tubular acidoses (RTAs) are selective inherited or acquired defects of the proximal (type 2 RTA), distal (type 1 RTA), or both (type 3 RTA) tubular functions, uncoupled to reduction of GFR. They may present with different clinical features, as listed in Table 3, but in all of them sAG is unchanged: for example in proximal RTA loss of bicarbonate is coupled to retention of chloride. In RTAs serum bicarbonate is low, both because of urinary loss and compensatory hyperventilation; PCO_2_ is low, *S*_dp_ is diminished and BE is negative; moreover, ammoniagenesis and NAE are reduced (Harris et al., 2023; Wang et al., 2024; Kunchur et al., 2024).

RTAs present a complex relationship with alterations of serum potassium levels. Potassium and ammonium compete for several renal ion transporters; thus, the reduction of ammoniagenesis in RTAs of types 1, 2, and 3 is associated with loss of potassium and hypokalemia. There is a fourth type of RTA, frequently observed in Addison's disease, in which hyperkalemia is present and demands increased potassium excretion, thus causing decreased ammoniagenesis, increased NH_4_^+^ reabsorption, and decreased NAE; in this case the kidney is often healthy, and the disease has an endocrine cause. Conversely Cushing's disease is often associated with hypokalemia and metabolic alkalosis (Fraser, 1984).

### 4.4 Nonrenal metabolic acidoses

Metabolic disturbances that produce excess of nonvolatile acids (e.g. ketoacids, lactic acid) cause HAGMA. In these cases PCO_2_ and bicarbonate are low because of compensatory hyperpnea, while the urine is strongly acidic and renal ammoniagenesis, urinary excretion of ammonia, and NAE are strongly increased. For example, in a case of diabetic ketoacidosis reported by Schoolwerth, the urinary excretion of ammonia amounted to 186 mmol/day, some 6‐fold higher than normal (Schoolwerth, 1991). *S*_dp_ is reduced and BE is negative.

Extra‐renal loss of bicarbonate is observed for example in severe diarrhea (e.g., cholera), and is made worse by the compensatory hyperventilation. Since in these conditions protein loss is typically modest, the sAG increases due to the increased relative contribution of proteins to the sum of negative charges, and the condition is that of HAGMA, with decreased PCO_2_ and bicarbonate, decreased *S*_dp_ and negative BE.

Dilution acidosis is an iatrogenic condition due to infusion of large volumes of isotonic saline that may resemble MA. It is caused by dilution of all nonvolatile buffer components of blood, while PCO_2_ remains normal, thus causing acidosis.

Table 3 summarizes some examples of renal and extra‐renal MAs and clearly shows that these conditions present very similar blood gas analyses (except for the sAG), but very different urinalyses, strengthening the notion that in metabolic disturbances of the acid–base equilibrium the blood gas analysis is usefully complemented by urinalysis (Uribarri et al., 2022).

### 4.5 Metabolic alkalosis

Metabolic alkalosis is caused by excess loss of nonvolatile acid. Respiratory compensation occurs by reducing the ventilation and consequently the elimination of CO_2_, but this mechanism is limited by the O_2_ requirements. One may again distinguish renal and extra‐renal conditions; however, metabolic alkaloses of renal origin, such as the genetically determined Bartter and Gitelman syndromes (salt losing tubulopathies; see Table 3), are rare; they may cause hypokalemia and hypochloremia in addition to the alkalosis (Fulchiero & Seo‐Mayer, 2019; Kamel et al., 1998). A typical example of an extra‐renal metabolic alkalosis is provided by prolonged vomiting: the stomach produces hydronium ions via carbonic anhydrase and secretes them together with chloride in the gastric juice, while retaining bicarbonate ions in the circulation. If vomiting occurs, the loss of acidic gastric juice forces the stomach to produce more, thus releasing more bicarbonate in the bloodstream; the final result is a metabolic alkalosis with increased pH, increased serum bicarbonate, positive BE and SBE, and a compensatory increase of PCO_2_. The kidney may also participate in the compensation mechanisms by excreting bicarbonate.

A clinical example is the following: blood gas analysis: pH 7.59; PCO_2_ 33 mmHg; BE 10 mEq/L; HCO_3_^−^ 31.7 mM. Urine analysis: pH 6.5; Cl^−^ 118 mEq/24 h; Na^+^ 110 mEq/24 h; K^+^ 20 mEq/24 h; uAG 12 mEq/24 h (urinary ammonium not available).

## 5 CONCLUSIONS

Powerful explanatory concepts were developed to study the respiratory and renal contributions to acid–base balance: e.g. base excess, Winter's rules, the net acid excretion. However, the two fields of pneumology and nephrology have largely resisted cross‐fertilization, and it is commonly observed in the literature that blood gas analyses are interpreted in the absence of information on the urine composition and vice versa. It appears highly desirable that these two fields of clinical investigation find a synthesis. Our analysis strongly suggests that for a complete and reliable interpretation of acid–base imbalances both the blood gas analysis and the urine analysis are required, especially in the cases of metabolic and mixed conditions; moreover the 24 h urine analysis should routinely include, besides pH and common electrolytes, ammonium and/or NAE. We strongly concur with Kirschbaum et al. (1999), and with Uribarri et al. (2022) who advocate the direct measurement of urinary ammonium instead of its estimate from uAG.

## AUTHOR CONTRIBUTIONS

**Andrea Bellelli:** Conceptualization. **Vincenzo Graziano:** Investigation. **Antonio Angeloni:** Formal analysis; methodology. **Paolo Mené:** Formal analysis; methodology.

## FUNDING INFORMATION

We received no funding for this research.

## ETHICS STATEMENT

This work does not involve human subjects nor makes use of laboratory animals.

## CONSENT

This work does not involve human subjects.

## Data Availability

A computer program implementing the model described by Bellelli (2025), is publicly available at: https://www.andreabellelli.it/html/didattica/Fcourse/L15_Electrolytes/BloodBuffers.php.
